# Diagnostic Accuracy of FDG PET-CT in Lymph Nodal Staging of Lung Cancer

**DOI:** 10.7759/cureus.77880

**Published:** 2025-01-23

**Authors:** Iram Sagheer Khan, Saira Mashkoor, Seema Shafiq, Faiza Amber, Raima Kaleemi, Sadaf Nausheen, Pinkey Kumari, Atif A Hashmi

**Affiliations:** 1 Radiology, United Medical and Dental College, Karachi, PAK; 2 Radiology, Altnagelvin Hospital, Galway, IRL; 3 Radiology, Jinnah Postgraduate Medical Centre, Karachi, PAK; 4 Radiology, Dow University of Health Sciences, Karachi, PAK; 5 Nuclear Medicine, Jinnah Postgraduate Medical Centre, Karachi, PAK; 6 Internal Medicine, Hamdard College of Medicine and Dentistry, Karachi, PAK; 7 Pathology, Liaquat National Hospital and Medical College, Karachi, PAK

**Keywords:** computed tomography, diagnostic accuracy, lung cancer, lymph nodal staging, positron emission tomography

## Abstract

Background

Lymph node staging is a critical component in managing lung cancer, as it determines prognosis and guides treatment decisions. Fluorodeoxyglucose (FDG) positron emission tomography-computed tomography (PET-CT) has emerged as a highly sensitive and specific imaging modality for evaluating nodal involvement, surpassing conventional imaging techniques. Therefore, this study evaluated the diagnostic accuracy of PET-CT in lymph nodal staging of lung cancer, using histopathology as the gold standard.

Methodology

This cross-sectional study was conducted at the Radiology and PET Scan Department, Jinnah Postgraduate Medical Centre (JPMC), Karachi, over six months, from January 28, 2021, to July 28, 2021, using a non-probability consecutive sampling. It included 127 biopsy-proven lung cancer patients aged 21-80 years. FDG PET-CT scans were evaluated by an experienced radiologist, with findings compared against histopathology results after surgery and biopsy. Using IBM SPSS Statistics for Windows, Version 22 (Released 2013; IBM Corp., Armonk, New York, United States), data analysis included calculating the sensitivity, specificity, and predictive values of FDG PET-CT for nodal metastasis, with histopathology as the gold standard.

Results

The study findings showed that out of 127 lung cancer patients, with a mean age of 54.14 ± 10.41 years, 99 (78.0%) were male, and 111 (87.4%) were aged >40 years. Symptoms persisted for a mean of 12.04 ± 6.82 weeks, and 88 (69.30%) had standardized uptake value (SUV)>3.0. Nodal staging showed N1 nodes in 64 (50.40%), N2 in 37 (29.10%), and N3 in 26 (20.50%). PET-CT detected nodal metastasis in 89 (70.18%) of cases, with histopathology confirming metastasis in 95 (74.88%). PET-CT achieved a sensitivity of 91.50%, specificity of 93.75%, positive predictive value (PPV) of 97.75%, and negative predictive value (NPV) of 78.95%. The overall accuracy of PET-CT was 92.13%, with a strong correlation to histopathology, emphasizing its reliability in staging nodal metastasis.

Conclusion

This study concluded the high diagnostic accuracy of PET-CT in detecting nodal metastasis in lung cancer, with an overall accuracy of 92.13%. PET-CT demonstrated excellent sensitivity (91.50%) and specificity (93.75%). These results confirm PET-CT as a reliable imaging modality for lymph nodal staging.

## Introduction

Lung cancer is the most prevalent cancer globally and accounts for the highest number of cancer-related deaths. As reported by the World Health Organization (WHO), it remains the leading cause of cancer fatalities worldwide [1]. According to WHO in 2020, lung cancer claimed 1.80 million lives across the globe [2]. Unfortunately, Pakistan lacks a centralized national cancer registry, which poses challenges in monitoring the annual incidence of various cancer types. Nevertheless, several cities maintain cancer registries, including the Punjab Cancer Registry in Lahore, the Karachi Cancer Registry, and the Pakistan Atomic Energy Commission Cancer Registry (PAEC) in Islamabad [3]. 

The management and prognosis of lung cancer are influenced by its histological classification and the stage at which it is diagnosed. Lung cancer is primarily categorized into two types: small-cell lung cancer (SCLC) and non-small-cell lung cancer (NSCLC) [4]. NSCLC progresses more slowly compared to SCLC and is further subdivided into three histopathological subtypes: squamous cell carcinoma, adenocarcinoma, and large cell carcinoma [5]. Lung adenocarcinoma is the most prevalent form of lung cancer, accounting for approximately 40% of all cases. It typically develops in the peripheral regions of the lungs, originating from small airway epithelial cells responsible for secreting mucus and other substances [6]. Squamous cell carcinoma (SCC) is the second most common type of lung cancer, following adenocarcinoma. It arises from the bronchial surface epithelium, with tumor cells exhibiting a squamous appearance similar to epidermal cells. Among NSCLCs, SCC is most strongly associated with tobacco smoking. It typically forms in the central regions of the larger bronchi, often metastasizing to locoregional lymph nodes early in its progression. However, it tends to spread beyond the thoracic cavity at a later stage compared to other major types of lung cancer [7].

Accurate staging is crucial for effectively managing patients with NSCLC, as it guides the selection of the most appropriate treatment approach and aids in predicting outcomes [8]. The tumor, node, metastasis (TNM) staging system for lung cancer was developed by the American Joint Commission on Cancer (AJCC) and the Union for International Cancer Control (UICC) [9]. This system classifies lung cancer based on the size and extent of the primary tumor, the involvement of lymph nodes, and the presence of distant metastases. Determining the TNM stage involves a combination of the patient’s medical history, physical examination, and findings from imaging and pathology reports provided by radiologists and pathologists [10]. The five-year survival rate for small, localized stage 1 NSCLCs treated with surgical resection ranges from approximately 70% to 90%. However, the majority of patients, around three-quarters are diagnosed at advanced stages, which are associated with a poor prognosis [11,12].

Staging NSCLC is a multidisciplinary effort involving imaging, endoscopic procedures, and surgical techniques. Accurate clinical staging of mediastinal lymph nodes (MLNs) is essential for identifying patients eligible for surgical resection, as MLN involvement is a critical prognostic factor in potentially resectable NSCLC. Patients with stage I, II, or III NSCLC without lymph node metastases (N0) or with ipsilateral hilar lymph node metastases (N1) are typically considered for surgical resection. In contrast, those with ipsilateral mediastinal lymph node metastases (N2) or contralateral mediastinal lymph node metastases (N3) are usually referred for chemoradiation therapy [13].

Positron emission tomography-computed tomography (PET-CT) using fluorodeoxyglucose (FDG) has been widely recognized and incorporated into numerous international guidelines as a non-invasive method for staging lung cancer [14]. FDG PET-CT labeled with fluorine-18 (F-18), a glucose analog, has become a standard tool in clinical practice for evaluating lung nodules and staging lung cancer. Due to its high diagnostic accuracy, FDG PET-CT is now routinely included in many clinical lung cancer guidelines [15,16]. FDG PET-CT plays a significant role in diagnosing and staging NSCLC. The maximum standard uptake value (SUVmax), a measure derived from FDG PET-CT, represents the activity in tissue per unit volume relative to the administered dose and body weight. Studies have shown that the SUVmax of primary tumors correlates with factors such as stage, nodal status, histological type, differentiation, and tumor progression in NSCLC patients. Moreover, a high SUVmax is associated with worse prognostic outcomes [17].

While FDG PET-CT is valuable, histopathologic confirmation is required for treatments other than surgery in patients with PET-CT-positive lesions [15]. Notably, transthoracic biopsy, a common method for histological confirmation, carries a 20% risk of pneumothorax. This risk can delay further diagnostic or therapeutic procedures in cases of malignant lesions. FDG PET-CT, as a non-invasive imaging technique, may help minimize such risks for many patients with lung nodules, underscoring its importance in the diagnostic workflow [18].

Lung cancer is a significant health burden in Pakistan, with many cases diagnosed at advanced stages due to limited access to early detection and diagnostic tools. Accurate lymph nodal staging is essential for guiding appropriate treatment, yet invasive methods like histopathology are often challenging to perform in resource-limited settings. PET-CT, as a non-invasive and efficient imaging modality, holds the potential to address this gap by improving diagnostic accuracy and aiding in timely decision-making. Establishing its reliability in the local population is crucial for integrating it into routine clinical practice in Pakistan, where healthcare resources and patient accessibility vary widely. Therefore, this study evaluated the diagnostic accuracy of PET-CT in lymph nodal staging of lung cancer, using histopathology as the gold standard.

## Materials and methods

This cross-sectional study was conducted at the Radiology and PET Scan Department, Jinnah Post Graduate Medical Center (JPMC), Karachi, using a non-probability consecutive sampling technique. The Ethical Review Board of Jinnah Post Graduate Medical Center (F.2-81/2021-GENL/56903/JPMC) approved the study. The duration of the study was about six months, from 28th January 2021 to 28th July 2021. This study included 127 patients of both genders with biopsy-proven cases of lung cancer as informed by patient history and shown by previous hospital records, aged 21-80 years. Whereas, pregnant patients and biopsy-proven cases of lung cancer treated with radiotherapy and/or chemotherapy were excluded from the study.

After the synopsis was approved, all patients meeting the selection criteria were included in the study. Verbal informed consent was obtained from each participant. The researcher collected patient demographics and clinical history. FDG PET-CT scans were conducted at the Radiology and PET Scan Department of JPMC Karachi. The scans were evaluated on a console by a senior radiologist with over five years of post-fellowship experience, assisted by the researcher. The TNM staging system for lung cancer is a standardized method used to classify the extent of the disease based on three factors: tumor size and extent (T), regional lymph node involvement (N), and distant metastasis (M). The T category describes the size and local spread of the tumor. T1 represents tumors ≤3 cm, subdivided into T1a (≤1 cm), T1b (>1 cm but ≤2 cm), and T1c (>2 cm but ≤3 cm). T2 includes tumors >3 cm but ≤5 cm, with further division into T2a (>3 cm but ≤4 cm) and T2b (>4 cm but ≤5 cm), while T3 covers tumors >5 cm but ≤7 cm or those involving nearby structures such as the chest wall. T4 describes tumors >7 cm or those invading critical structures like the heart or trachea. The N category indicates regional lymph node involvement, where N0 means no lymph node involvement, N1 refers to ipsilateral peribronchial or hilar nodes, N2 involves ipsilateral mediastinal or subcarinal nodes, and N3 indicates contralateral lymph node involvement, including the supraclavicular region. The M category addresses distant metastasis, with M0 representing no metastasis and M1 indicating distant spread to organs [19]. Lesions identified as positive for nodal metastasis of lung cancer were referred for surgery and biopsy. These patients were subsequently followed, and their histopathological findings were documented. The clinical history and FDG PET-CT scan findings for each patient were recorded in the proforma, along with the final histopathological diagnosis.

The recommended FDG dosage typically ranges from 3.7-5.2 MBq/kg, with a resting uptake period of around 60 minutes. Imaging should cover the skull base to mid-thigh, using a PET-CT scanner with appropriate acquisition parameters, including 3-5 mm slice thickness and iterative reconstruction techniques for optimal image quality. In PET-CT interpretation, a lymph node is considered "positive" for metastasis based on factors such as increased FDG uptake with a standardized uptake value (SUV) typically above 2.5-3.5, abnormal nodal size greater than 10 mm in short-axis diameter, and irregular shape or loss of fatty hilum on CT images.

For histopathological diagnosis of nodal metastasis, samples are commonly obtained through endobronchial ultrasound/transbronchial needle aspiration (EBUS/TBNA), mediastinoscopy, or surgical resection, followed by fixation in formalin and processing for H&E staining and immunohistochemistry. Diagnostic criteria include the presence of atypical epithelial cells inside the nodal capsule with/without extracapsular extension into the fat. In challenging cases, immunohistochemistry was applied to identify metastatic carcinoma. The staging was done as per AJCC guidelines.

Data was analyzed using IBM SPSS Statistics for Windows, Version 22 (Released 2013; IBM Corp., Armonk, New York, United States). The mean and standard deviation were calculated for quantitative variables like age, duration of symptoms, and value of SUV on FDG PET. Frequency and percentage were evaluated for nodal stage of lung cancer (N1, N2, N3), gender, and presence of nodal metastasis on FDG PET-CT and histopathology. A chi-square test was used to compare the lymph node characteristics and metastasis between age groups and gender. The diagnostic accuracy of FDG PET-CT in the diagnosis of nodal metastasis in lung cancer was determined in terms of sensitivity, specificity, positive predictive value, and negative predictive value, taking the gold standard of histopathology using a 2 x 2 table. A p-value of less than 0.05 was considered statistically significant. 

## Results

The study included a total of 127 patients with lung cancer. The mean age of the patients was 54.14 ± 10.41 years. Around 16 (12.6%) patients were aged ≤40 years, while the majority, 111 (87.4%) patients, were aged >40 years. Regarding gender distribution, 99 (78.0%) patients were male, and 28 (22.0%) were female, indicating a higher prevalence of lung cancer among males, as presented in Table 1.

**Table 1:** Demographic details of the patients with lung cancer (n=127)

Variables		Mean ± SD n(%)
Age (years)		54.14 ± 10.41
Age group	≤40 years	16 (12.60%)
	>40 years	111 (87.40 %)
Gender	Male	99 (78.0%)
	Female	28 (22.0%)

The mean duration of symptoms was 12.04 ± 6.82 weeks, with 84 (66.10%) of patients experiencing symptoms for more than eight weeks. The mean SUV value was 3.34 ± 1.00, with 88 (69.30%) of patients presenting an SUV value >3.0. The mean size of lymph nodes was 2.19 ± 0.86 cm, and more than half (66, 52.0%) had lymph nodes larger than 2.0 cm. Regarding nodal staging, 64 (50.40%) of patients had N1 nodes, while N2 and N3 nodes were observed in 37 (29.10%) and 26 (20.50%), respectively, as presented in Table 2.

**Table 2:** Clinical and diagnostic characteristics of patients with lung cancer SUV: standardized uptake value

Variables		Mean ± SD, n(%)
Duration of symptoms (weeks)		12.04 ± 6.82
Duration of symptoms (weeks)	≤8	43 (33.90%)
	>8	84 (66.10%)
SUV		3.34 ± 1.00
SUV	≤3.0	39 (30.70%)
	>3.0	88 (69.30%)
Size of the lymph node (cm)		2.19 ± 0.86
Size of the lymph node (cm)	≤2.0	61 (48.0%)
	>2.0	66 (52.0%)
Nodal staging	N1	64 (50.40%)
	N2	37 (29.10%)
	N3	26 (20.50%)

**Figure 1:**
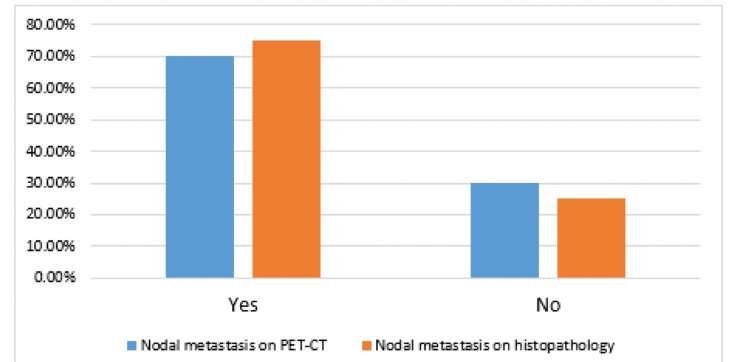
The presence of nodal metastasis on PET-CT and histopathology PET-CT: positron emission tomography-computed tomography

PET-CT identified nodal metastasis in 89 (70.18%) of cases, while histopathology confirmed nodal metastasis in 95 (74.88%) of patients, as presented in Figure 1.

The comparison of lymph node characteristics and metastasis between age groups showed no statistically significant differences across most variables. Lymph node size ≤2.0 cm was observed in seven (43.8%) patients under 40 years and 54 (48.6%) over 40 years (p = 0.714). Nodal staging showed a higher proportion of N1 in younger patients (10, 62.5% vs. 54, 48.6%), but the difference was not significant (p = 0.293). Nodal metastasis on PET-CT was more frequent in younger patients (14, 87.5% vs. 75, 67.6%) (p = 0.104). Similarly, nodal metastasis on histopathology was higher in patients under 40 years (15, 93.8% vs. 80, 72.1%), with an insignificant difference among them (p = 0.062), as presented in Table 3.

**Table 3:** The comparison of lymph node characteristics and metastasis between age groups PET-CT: positron emission tomography-computed tomography

Variables		Age group		
		< 40 years n(%)	>40 years n(%)	p-value
Size of the lymph node (cm)	≤2.0	7(43.8%)	54 (48.6%)	0.714
	>2.0	9(56.3%)	57 (51.4%)	
Nodal staging	N1	10 (62.5%)	54 (48.6%)	0.293
	N2	2 (12.5%)	35 (31.5%)	
	N3	4 (25.0%)	22(19.8%)	
Nodal metastasis on PET-CT	Yes	14(87.5%)	75(67.6%)	0.104
	No	2(12.5%)	36(32.4%)	
Nodal metastasis on histopathology	Yes	15(93.8%)	80(72.1%)	0.062
	No	1(6.2%)	31(27.9%)	

The comparison of lymph node characteristics and metastasis between male and female patients showed no statistically significant differences across most variables. Lymph node size ≤2.0 cm was seen in 46 (46.5%) of males and 15 (53.6%) of females (p = 0.506). Nodal staging patterns were similar between genders (p = 0.440). Nodal metastasis on PET-CT was more frequent in females (23, 82.1% vs. 66, 66.7%) (p = 0.114). However, a statistically significant difference was observed in nodal metastasis on histopathology, with 70 (70.7%) of males and 25 (89.3%) of females showing positive results, while 29 (29.3%) of males and three (10.7%) of females were negative (p = 0.046), as presented in Table 4.

**Table 4:** The comparison of lymph node characteristics and metastasis between male and female patients PET-CT: positron emission tomography-computed tomography

Variables		Gender		
		Male n(%)	Female n(%)	p-value
Size of the lymph node (cm)	≤2.0	46(46.5%)	15 (53.6%)	0.506
	>2.0	53 (53.5%)	13 (46.4%)	
Nodal staging	N1	47 (47.5%)	17 (60.7%)	0.440
	N2	30 (30.3%)	7 (25.0%)	
	N3	22(22.2%)	4 (14.3%)	
Nodal metastasis on PET-CT	Yes	66(66.7%)	23(82.1%)	0.114
	No	33(33.3%)	5(17.9%)	
Nodal metastasis on histopathology	Yes	70(70.7%)	25(89.3%)	0.046
	No	29(29.3%)	3(10.7%)	

A cross-tabulation of PET-CT and histopathology results showed that PET-CT detected nodal metastasis in 87 of the 95 cases confirmed by histopathology, yielding two false positives and eight false negatives. Among the 32 patients without histopathological evidence of nodal metastasis, PET-CT correctly identified 30 as negative. This indicates a strong correlation between PET-CT and histopathology in detecting nodal metastasis, with high diagnostic accuracy, as presented in Table 5.

**Table 5:** Comparison of nodal metastasis detection by PET-CT and histopathology PET-CT: positron emission tomography-computed tomography

PET-CT	Histopathology		
	Present	Absent	Total
Present	87	2	89
Absent	8	30	38
Total	95	32	127

The diagnostic accuracy of PET-CT revealed an overall accuracy of 92.13%. PET-CT exhibited a sensitivity of 91.50% and a specificity of 93.75%. The positive predictive value (PPV) was 97.75%, indicating high reliability in confirming metastasis. The negative predictive value (NPV) was 78.95%, reflecting its moderate ability to exclude metastasis, as presented in Table 6.

**Table 6:** Overall diagnostic accuracy of PET-CT taking histopathology as the gold standard PET-CT: positron emission tomography-computed tomography

Variables	Accuracy	Sensitivity	Specificity	Positive predictive value	Negative predictive value
PET-CT vs. Histopathology as gold standard	92.13%	91.50%	93.75%	97.75%	78.95%

## Discussion

Accurate staging of NSCLC is crucial for deciding the best treatment approach for patients. FDG PET-CT is the most sensitive and precise method for detecting hilar and mediastinal lymph node (MLN) involvement. It is also the most reliable technique for identifying metastatic disease, whether local, regional, or distant [20]. Therefore, this study demonstrated the diagnostic accuracy of PET-CT in lymph nodal staging of lung cancer.

We had a higher proportion of men in our study cohort, which is higher than the expected rate of the worldwide prevalence of gender-based lung cancer. One of the reasons can be a selection bias, as the study involved a single public sector hospital and represents the lower socioeconomic sector of the population. Moreover, reliable data regarding the gender-based prevalence of lung cancer is lacking in Pakistan. Men generally have higher smoking rates than women, which is a significant risk factor for lung cancer and may have contributed to their greater participation in the study. Recruitment strategies may have inadvertently favored male participants, while occupational exposure and lifestyle factors could also play a role in the gender disparity.

This study assessed the diagnostic accuracy of 18F-FDG PET-CT, CT scan, and endobronchial ultrasound/transbronchial needle aspiration (EBUS/TBNA) in staging MLNs in NSCLC patients. An 18F-FDG PET-CT showed superior sensitivity and specificity compared to CT, particularly in histopathologically confirmed cases. EBUS/TBNA and mediastinoscopy had the highest overall accuracy (88.2% and 88.6%, respectively), while 18F-FDG PET-CT was less accurate (70.2%). Lymph nodes with SUVmax >3 were more likely true positives, highlighting the diagnostic utility of 18F-FDG PET-CT, though invasive methods remain more reliable [21]. The present study was inconsistent with the above-reported study and reported a significantly higher overall accuracy (92.13%) and specificity (93.75%) for PET-CT. It also highlights a superior positive predictive value (97.75%), indicating exceptional reliability in confirming metastasis.

Some studies have demonstrated that 18F-FDG PET-CT offers higher sensitivity and specificity compared to CT scanning for evaluating MLNs [22]. One study found that 18F-FDG PET-CT had a visual PET positivity that showed sensitivity, specificity, PPV, NPV, and accuracy of 72.4, 76.1, 30.1, 95.1, and 75.6, respectively, in detecting MLN metastases [23]. In contrast, the present study demonstrated significantly higher diagnostic accuracy, with sensitivity (91.50%), specificity (93.75%), and overall accuracy (92.13%) indicating superior performance. The PPV is particularly noteworthy at 97.75%, showcasing strong reliability in confirming metastases, though the NPV (78.95%) is slightly lower than in the above-reported study.

Similarly, another study evaluated the diagnostic accuracy of 18F-FDG PET-CT in detecting bone, lung, and lymph node metastases in newly diagnosed patients. The results showed 100% sensitivity and specificity for bone metastases, high specificity for lung metastases (96%-100%), and 100% sensitivity for lymph node involvement, though specificity ranged from 89% to 100%. These findings highlight the high diagnostic accuracy of 18F-FDG PET-CT for bone and lymph node metastases, with slightly reduced reliability for lung metastases due to limited data [24]. As far as the present study is concerned, nodal metastases in lung cancer reported slightly lower sensitivity (91.50%) and specificity (93.75%) but still indicated strong diagnostic accuracy. Its high PPV (97.75%) highlighted PET-CT's reliability in confirming metastases, though the moderate NPV (78.95%) showed reduced efficacy in excluding false negatives.

Another study assessed the accuracy of FDG PET-CT in characterizing unspecified lung nodules detected during lung cancer screening in high-risk individuals. Among 100 nodules evaluated, FDG PET-CT visual analysis showed high sensitivity (84%), specificity (95%), and accuracy (91%), with an area under the curve (AUC) of 0.893. While it effectively detected 31 malignant nodules, it yielded six false negatives. Using SUVmax thresholds of 1.5, 2, and 2.5 improved specificity (97%) but reduced sensitivity (to as low as 46%) and overall accuracy (to 78%). These results highlight FDG PET-CT’s effectiveness, particularly through visual analysis, in identifying malignancy in lung nodules during screening [25]. These findings were corroborated with the present study and revealed that PET-CT demonstrated high diagnostic accuracy in detecting nodal metastasis, with an overall accuracy of 92.13%. It correctly identified 87 of 95 histopathologically confirmed cases with two false positives and eight false negatives and accurately ruled out metastasis in 30 of 32 cases. Sensitivity was 91.50%, specificity 93.75%, and PPV 97.75%, indicating strong reliability in confirming metastasis, though the NPV was moderate at 78.95%. To address the clinical implications of moderate NPV as noted in our study, clinicians should consider using PET-CT as part of a multi-modal approach, combining it with biopsy or other imaging techniques like MRI, particularly in cases with suspicious clinical signs or when PET-CT results are inconclusive. In diverse clinical settings, PET-CT’s potential to guide early diagnosis, reduce unnecessary biopsies, and streamline treatment decisions could significantly enhance patient outcomes, especially when integrated into a comprehensive diagnostic and treatment workflow. To minimize these errors, strategies such as refining SUV threshold values, using alternative imaging techniques (e.g., MRI), and incorporating clinical correlation with histopathological findings are essential. Regular training for radiologists to recognize and differentiate between malignant and benign FDG uptake patterns and the use of advanced imaging software for more accurate interpretation could also reduce diagnostic errors, enhancing the overall reliability of PET-CT in lung cancer management. In addition, we also emphasize the importance of confirmatory or complementary tests (e.g., EBUS/TBNA, mediastinoscopy) in cases of negative PET-CT results.

Likewise, another study analyzed the diagnostic accuracy of 18F-FDG PET-CT and chest high-resolution computed tomography (HRCT) in differentiating malignant from benign pulmonary nodules (PNs). The sensitivity, specificity, and accuracy of HRCT, PET-CT, and their combination were 83.3%, 70%, 77.3%; 91.7%, 62.5%, 78.4%; and 95.8%, 75%, 86.4%, respectively. An SUVmax between 2.5 and 8.0 suggested the need for combined evaluation, as lesions in this range could be either benign or malignant. The study recommended using PET-CT combined with HRCT to enhance diagnostic accuracy, as PET-CT alone had a sensitivity of 83.3%, a specificity of 62.5%, and an accuracy of 79.2% [26]. These findings were inconsistent with the present study and indicated that PET-CT demonstrated high diagnostic accuracy in detecting nodal metastasis, with an overall accuracy of 92.13%.

A meta-analysis assessed the diagnostic performance of fibroblast activation protein inhibitors (FAPI) PET-CT for detecting lymph node metastases in lung cancer patients. The pooled sensitivity, specificity, and AUC for FAPI PET-CT were 88%, 94%, and 0.97, respectively, indicating excellent diagnostic accuracy. FAPI PET-CT outperforms standard FDG PET-CT in detecting lymph node metastases, making it a highly effective tool in lung cancer staging [27]. The present study did not support the above-mentioned study and indicated that PET-CT demonstrated high diagnostic accuracy in detecting nodal metastasis, with an overall accuracy of 92.13%. Sensitivity was 91.50%, specificity 93.75%, and PPV 97.75%, indicating strong reliability in confirming metastasis, though the NPV was moderate at 78.95%.

This study has a few limitations. First, it was conducted in a single-center setting, which may limit the generalization of the findings. Second, the sample size, though adequate, might not fully capture variability across diverse populations. As the study population is relatively homogenous (patients from a single geographic region), it may not capture the variability seen in other ethnic or demographic groups. This limits the applicability of the findings to diverse populations. Third, false negatives and false positives highlight the potential for diagnostic discrepancies with PET-CT, especially in cases with borderline SUV values or small lymph nodes. Future studies should consider multicenter designs with larger, more diverse cohorts to validate these findings. Additionally, combining PET-CT with advanced imaging techniques or molecular biomarkers may further enhance diagnostic accuracy and reduce false results. Additionally, using complementary diagnostic techniques, such as biopsy or MRI, alongside PET-CT could improve diagnostic accuracy and reduce false positives or negatives. Using PET-CT in conjunction with histopathological examination can confirm PET-CT results. Combining PET-CT with other diagnostic tools, such as MRI, biopsy, and ultrasound, can help validate PET-CT findings and reduce the risk of false negatives or positives. However, our study did not focus on MRI and biopsy. However, our study underscores the role of PET-CT in the staging of lung cancer, especially in resource-limited countries, where more highly advanced techniques and procedures are not available. Early diagnosis of lymph node metastasis in lung cancer with timely management can make a huge impact on the ultimate outcome of these patients. 

## Conclusions

This study concluded the high diagnostic accuracy of PET-CT in detecting nodal metastasis in lung cancer, with an overall accuracy of 92.13%. PET-CT demonstrated excellent sensitivity (91.50%) and specificity (93.75%), along with a strong positive predictive value (97.75%). These results confirm PET-CT as a reliable imaging modality for lymph nodal staging. However, the moderate negative predictive value (78.95%) suggests a need for a cautious interpretation of negative results. PET-CT remains an invaluable tool in guiding clinical decision-making for lung cancer management.
